# Supplementary material for: Efficacy of simultaneous VEGF‐A/ANG‐2 neutralization in suppressing spontaneous choroidal neovascularization
Source: EMBO Mol Med. 2019 Apr 30;11(5):e10204. doi: 10.15252/emmm.201810204 (PMC6505683; doi:10.15252/emmm.201810204)

Appendix Table of Contents

1) **Appendix Figure Legends S1 – S3**
2) **Appendix Figures S1-S3**

1) Appendix Figure Legends S1 – S3

Appendix Figure Legend S1. Dual neutralization of VEGF-A and ANG-2 in JR5558 mice reduced the number of TUNEL-positive apoptotic nuclei in the photoreceptor layer.

A Bar/scatter graph of the number of TUNEL-positive cells in the retina of mice treated with 10 mg/kg IgG control, 5 mg/kg anti-VEGF-A, 5 mg/kg anti-ANG-2, 10 mg/kg anti-VEGF-A/ANG-2 or left untreated. Data shown as mean ± SEM with n = 10 animals per group and * denotes all significant changes after one-sided ANOVA and Tukey's multiple t-test (Tukey-Kramer HSD). Anti-VEGF-A/ANG-2 (**, P = 0.007) and Anti-ANG-2 (*, P = 0.011) are significantly different from IgG control.

B Annotated images of representative examples of retina TUNEL staining of untreated (top left), IgG control (top middle;10 mg/kg), anti-VEGF-A (bottom left; 5 mg/kg), anti-ANG-2 (bottom middle; 5 mg/kg), and anti-VEGF-A/ANG-2 (bottom right; 10 mg/kg). For better visualization images were converted to black/white modus using PhotoShop CS6 with contrast enhancement applied equally to all images and non-tissue background was removed using Lasso tool. Scale bars = 500 µm. SEM, standard error of the mean; ANOVA, analysis of variance.

Appendix Figure Legend S2. Coordinates of Iba1-Positive Cells on RPE/Choroid Flat Mounts

XY-coordinates of identified Iba1-positive cells per RPE/choroid were exported to GraphPad Prism and displayed as scatter plots to visualize distribution and clumping.

Appendix Figure Legend S3. Thumbnail images of annotated retinal flat mounts (TUNEL) and coordinates.

XY-coordinates of TUNEL-positive cells per retina were exported to GraphPad Prism and displayed as scatter plots to visualize distribution.

Appendix Figure S1

A)

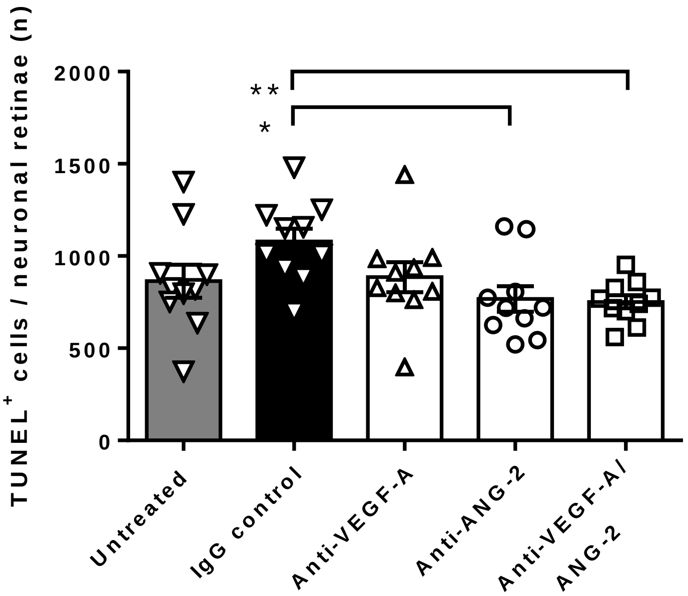

B)

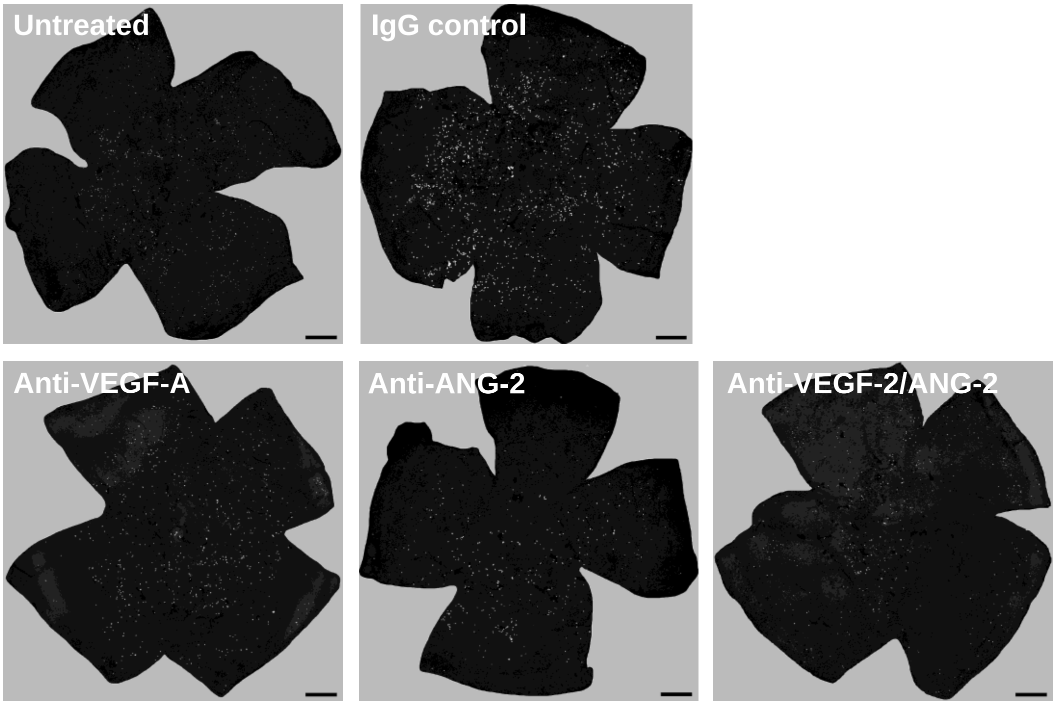

<u>APPENDIX FIGURE S2 - COORDINATES OF IBA1-POSITIVE CELLS ON RPE/CHOROID FLAT MOUNTS</u>

<u>A)</u> Group: Untreated

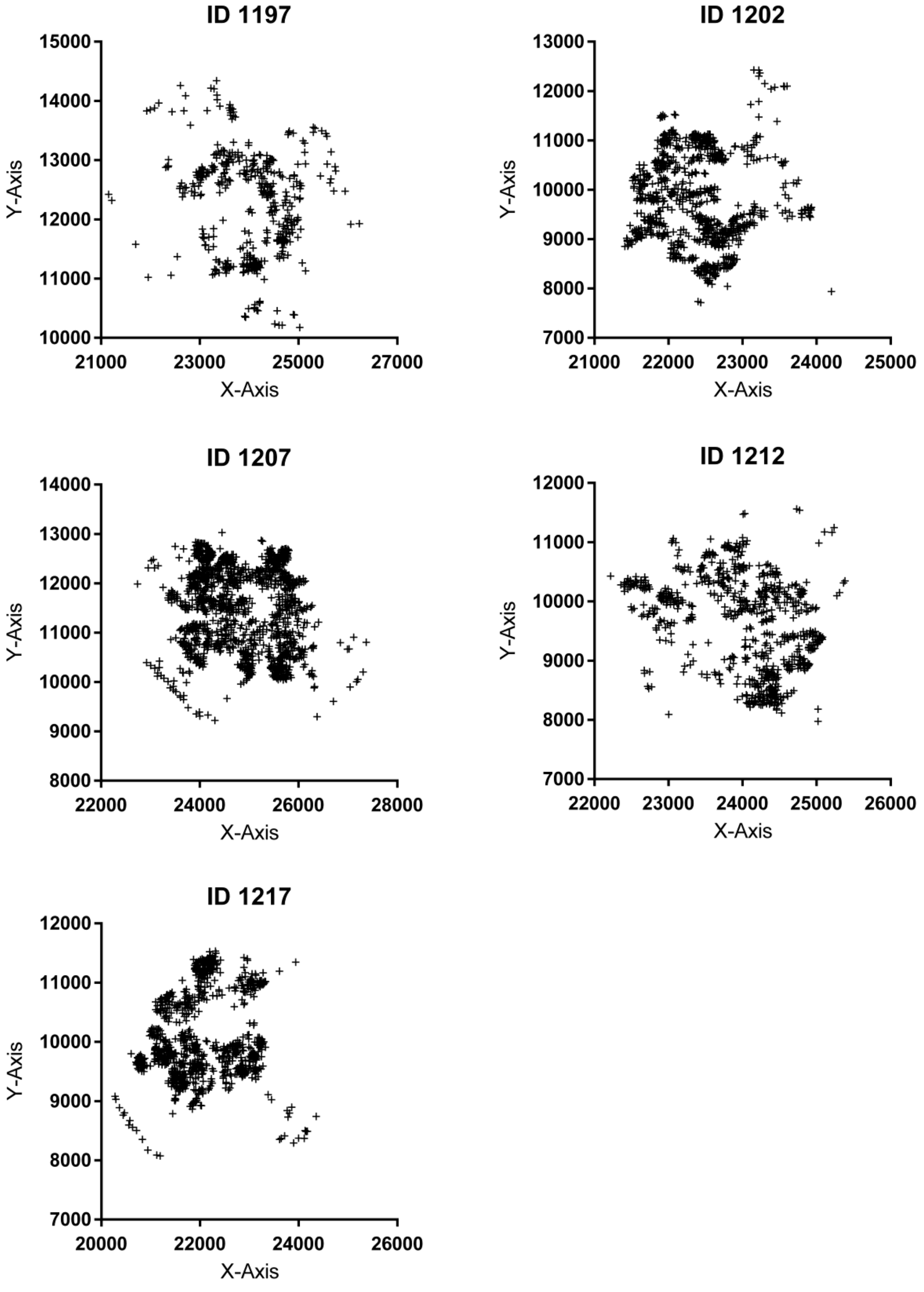

<u>APPENDIX FIGURE S2 - COORDINATES OF IBA1-POSITIVE CELLS ON RPE/CHOROID FLAT MOUNTS</u>

<u>B)</u> Group: IgG control

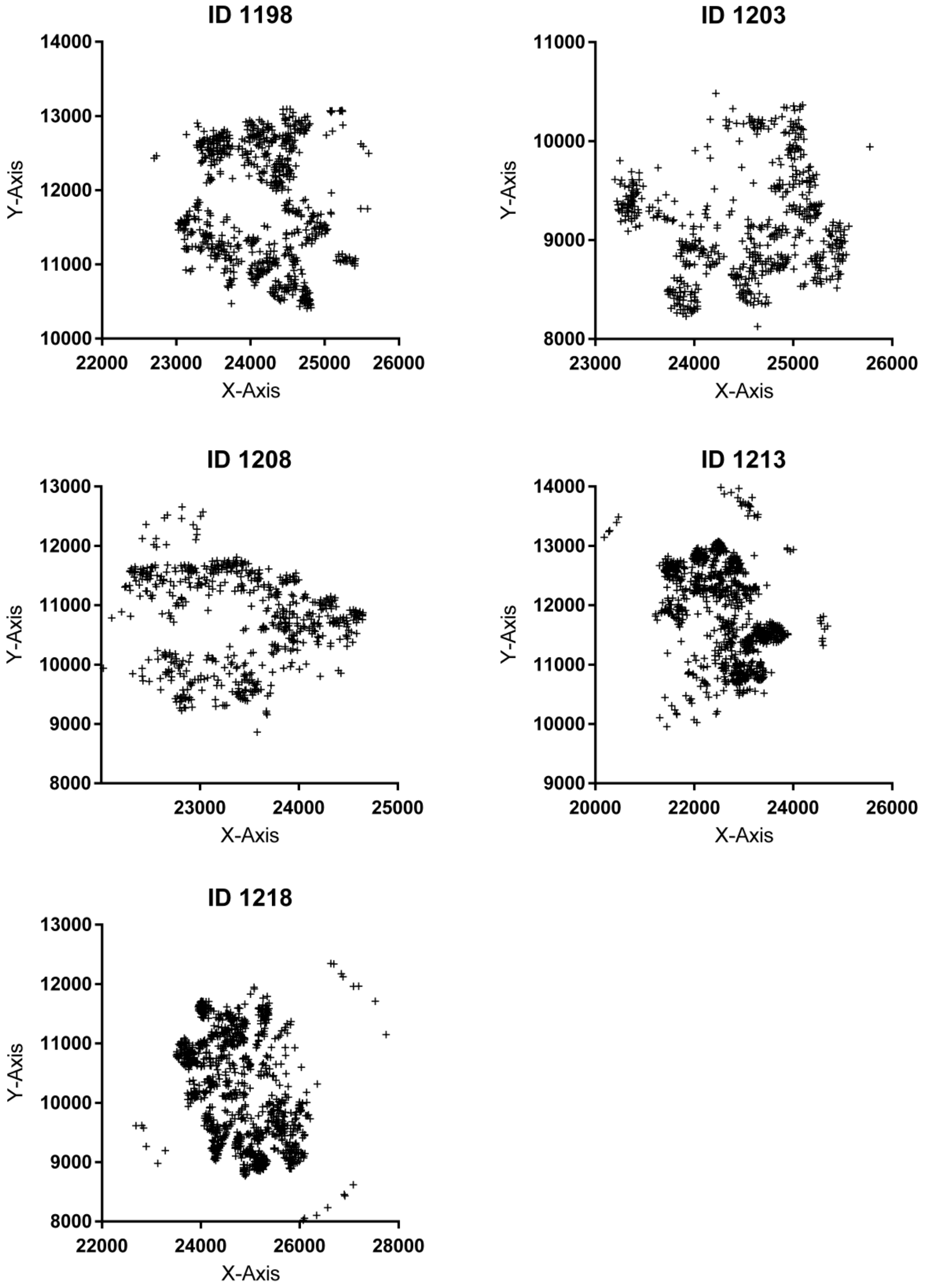

APPENDIX FIGURE S2 - COORDINATES OF IBA1-POSITIVE CELLS ON RPE/CHOROID FLAT MOUNTS

C) Group: Anti-VEGF-A

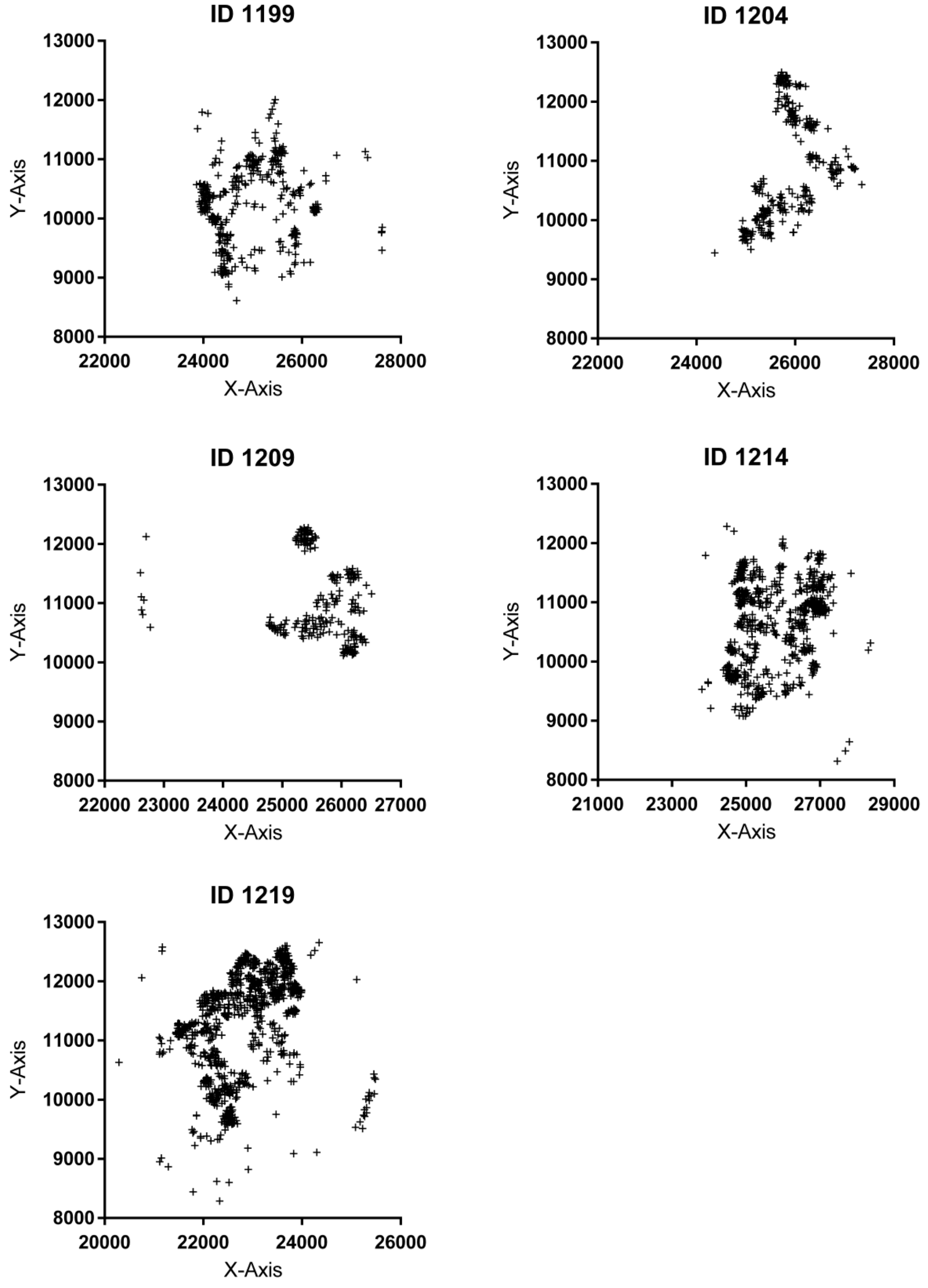

APPENDIX FIGURE S2 - COORDINATES OF IBA1-POSITIVE CELLS ON RPE/CHOROID FLAT MOUNTS

D) Group: Anti-ANG-2

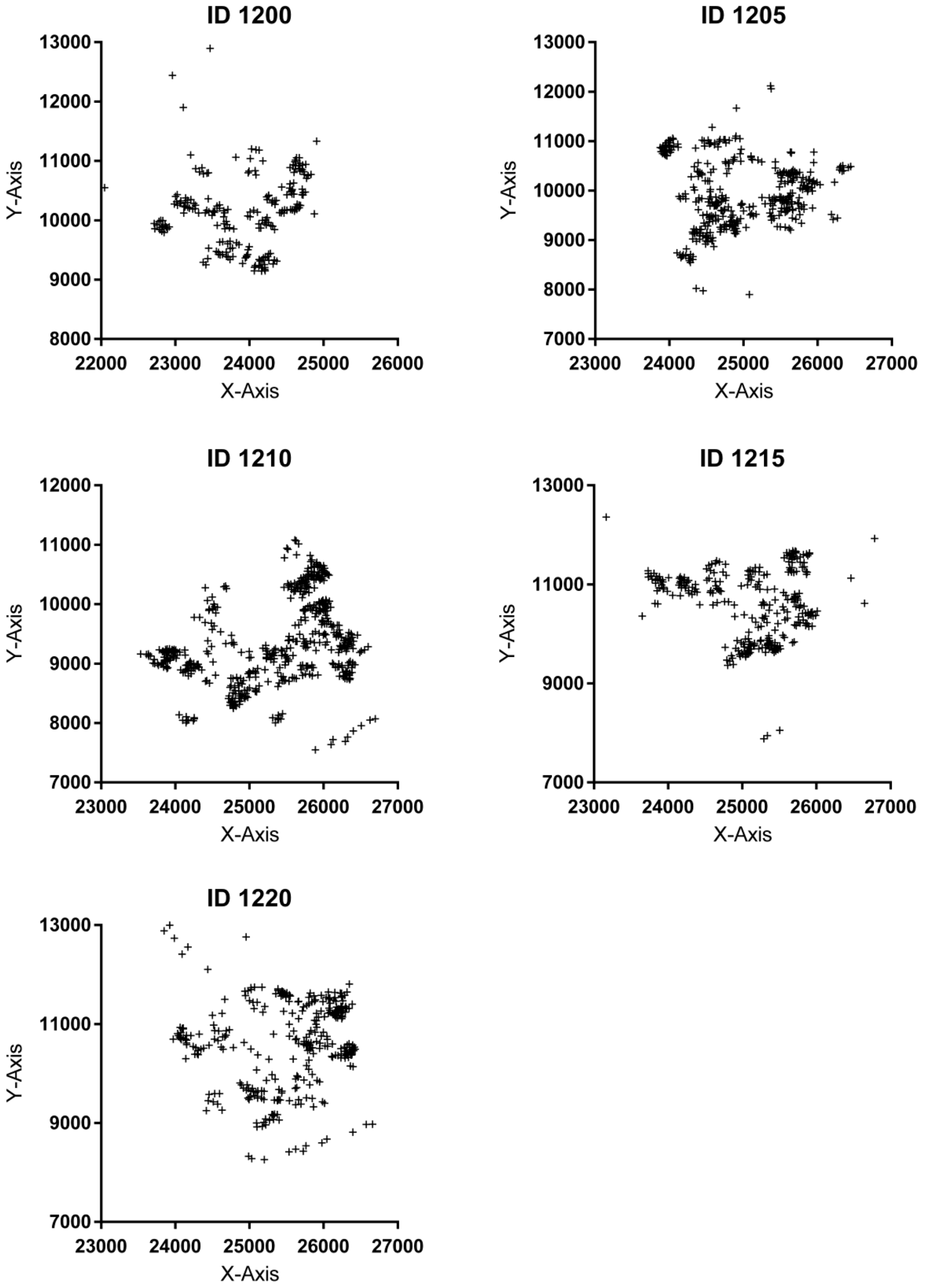

APPENDIX FIGURE S2 - COORDINATES OF IBA1-POSITIVE CELLS ON RPE/CHOROID FLAT MOUNTS

E) Group: anti-VEGF-A/ANG-2

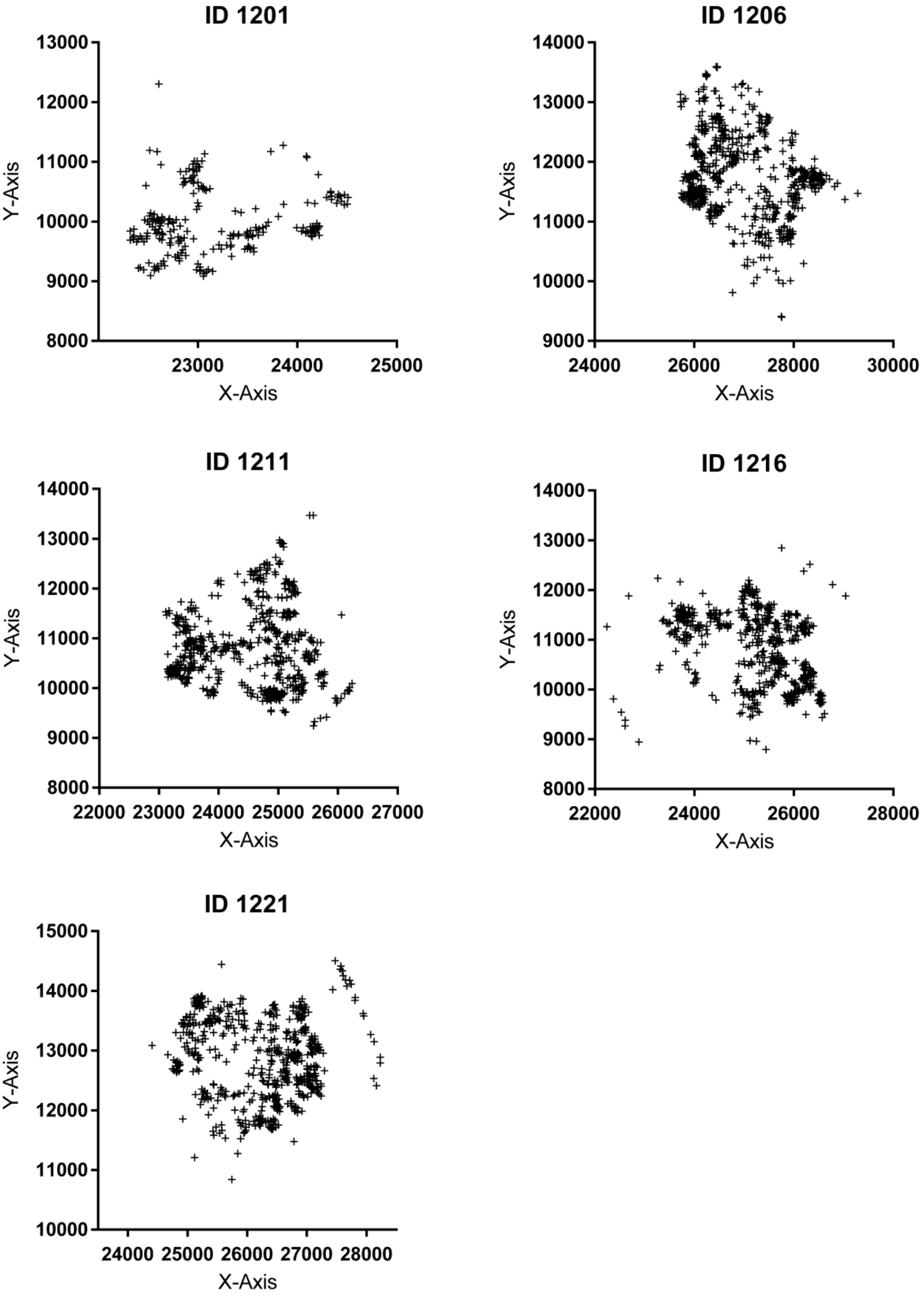

APPENDIX FIGURE S3 - THUMBNAIL IMAGES OF ANNOTATED RETINAL FLAT MOUNTS (TUNEL) AND COORDINATES

A) Group: Untreated

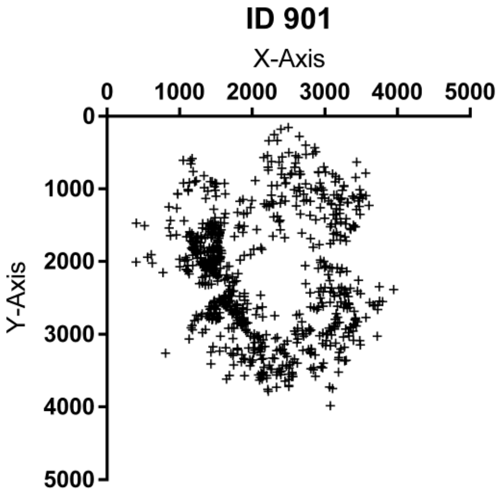

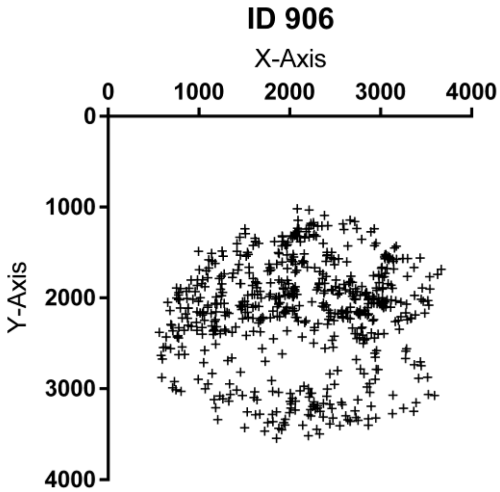

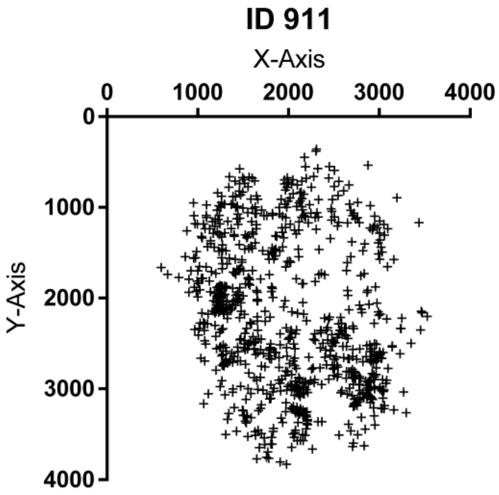

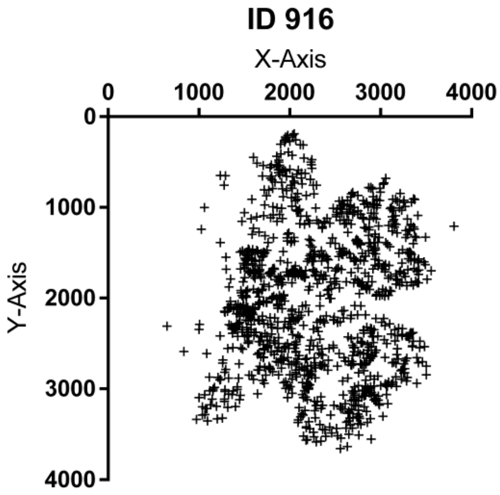

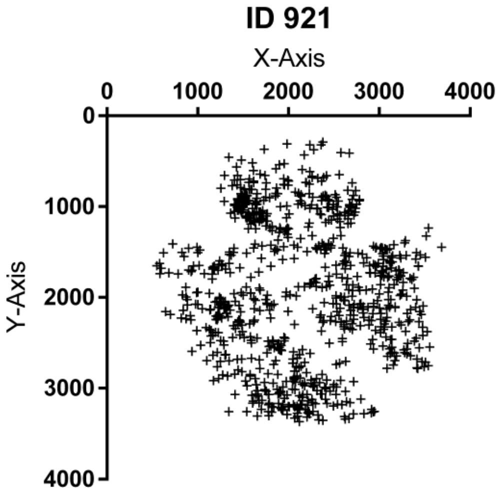

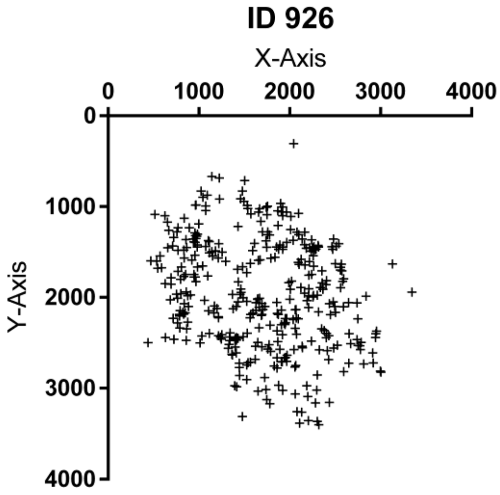

APPENDIX FIGURE S3 - THUMBNAIL IMAGES OF ANNOTATED RETINAL FLAT MOUNTS (TUNEL) AND COORDINATES

A) Group: Untreated

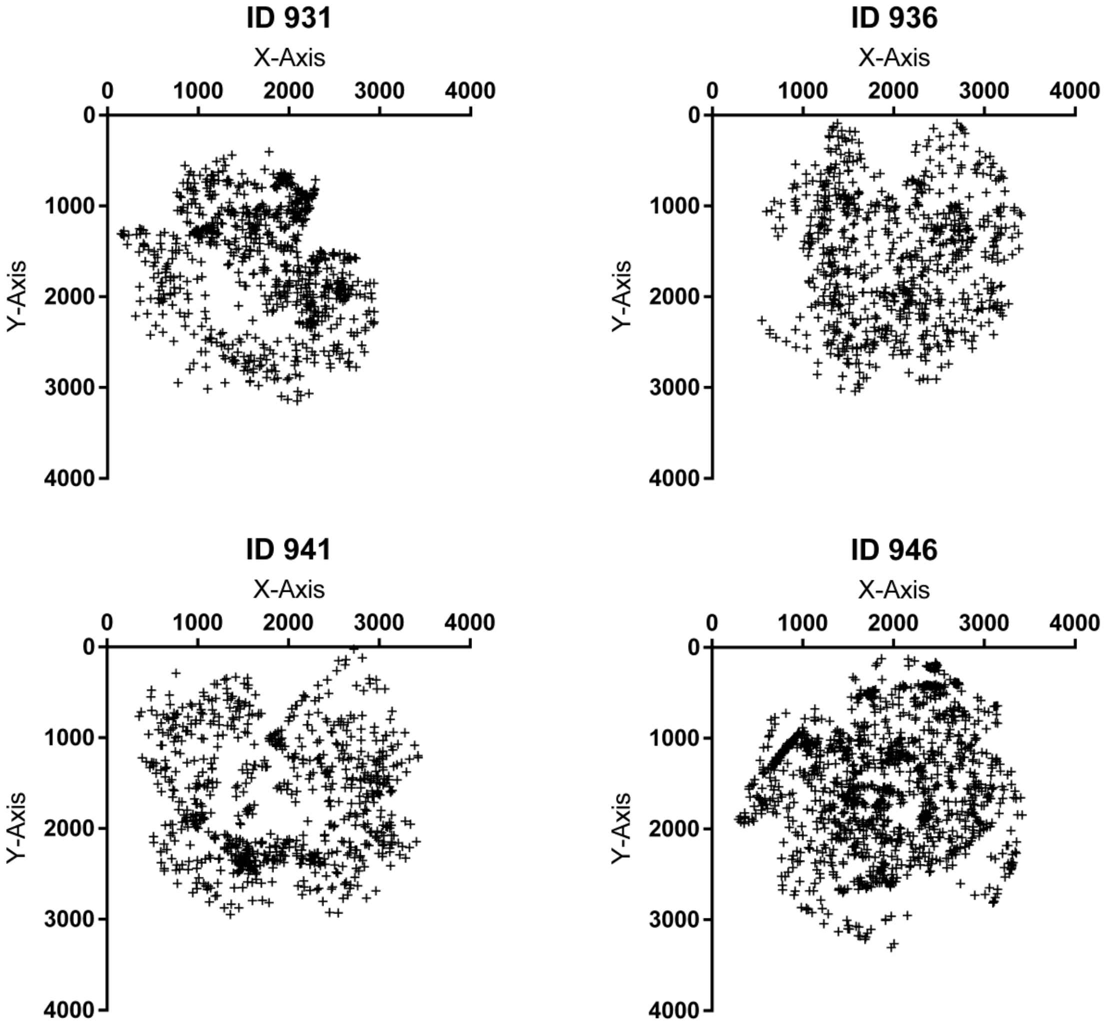

APPENDIX FIGURE S3 - THUMBNAIL IMAGES OF ANNOTATED RETINAL FLAT MOUNTS (TUNEL) AND COORDINATES

B) Group: IgG control

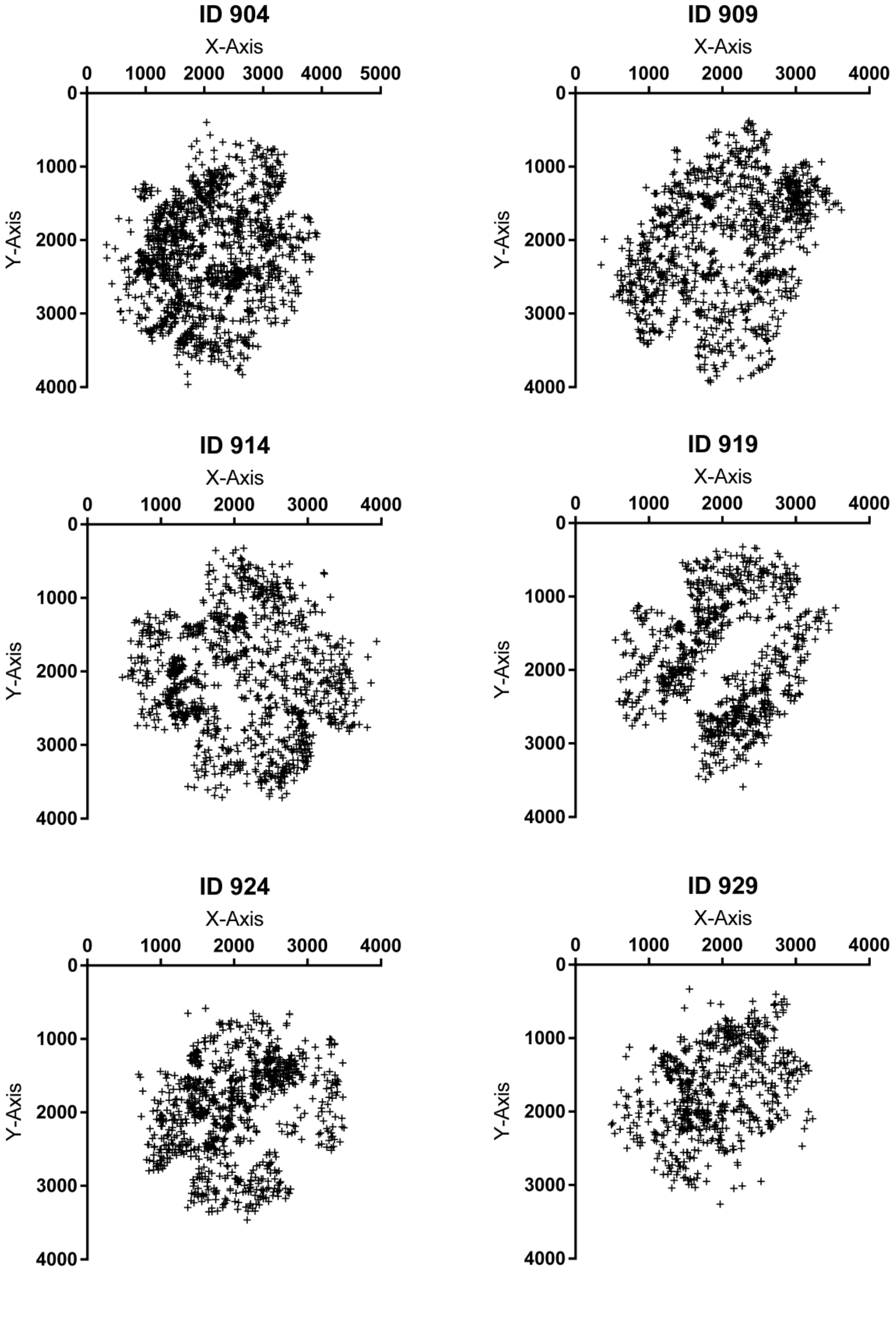

<u>APPENDIX FIGURE S3 - THUMBNAIL IMAGES OF ANNOTATED RETINAL FLAT MOUNTS (TUNEL) AND COORDINATES</u>

<u>B)</u> Group: IgG control

ID 934

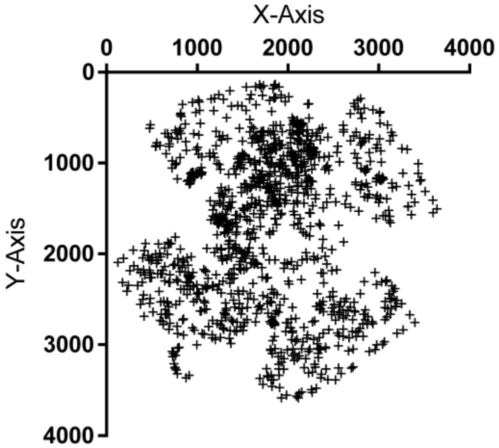

ID 939

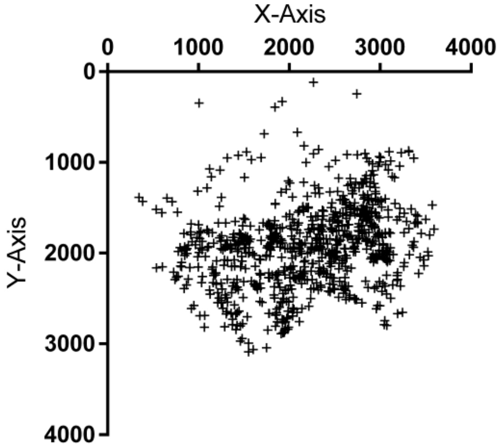

ID 944

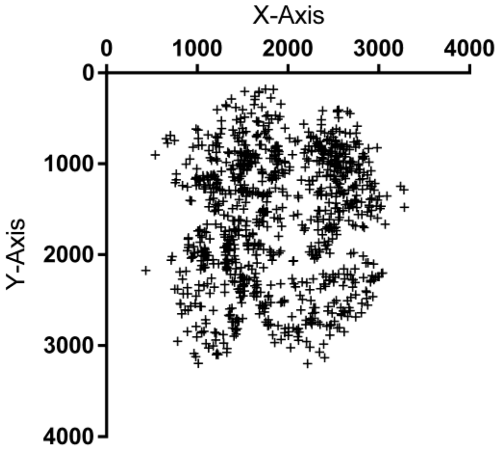

ID 949

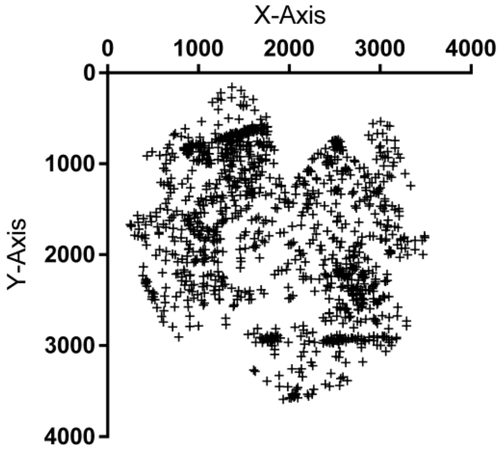

<u>APPENDIX FIGURE S3 - THUMBNAIL IMAGES OF ANNOTATED RETINAL FLAT MOUNTS (TUNEL) AND COORDINATES</u>
<u>C)</u> Group: Anti-VEGF-A

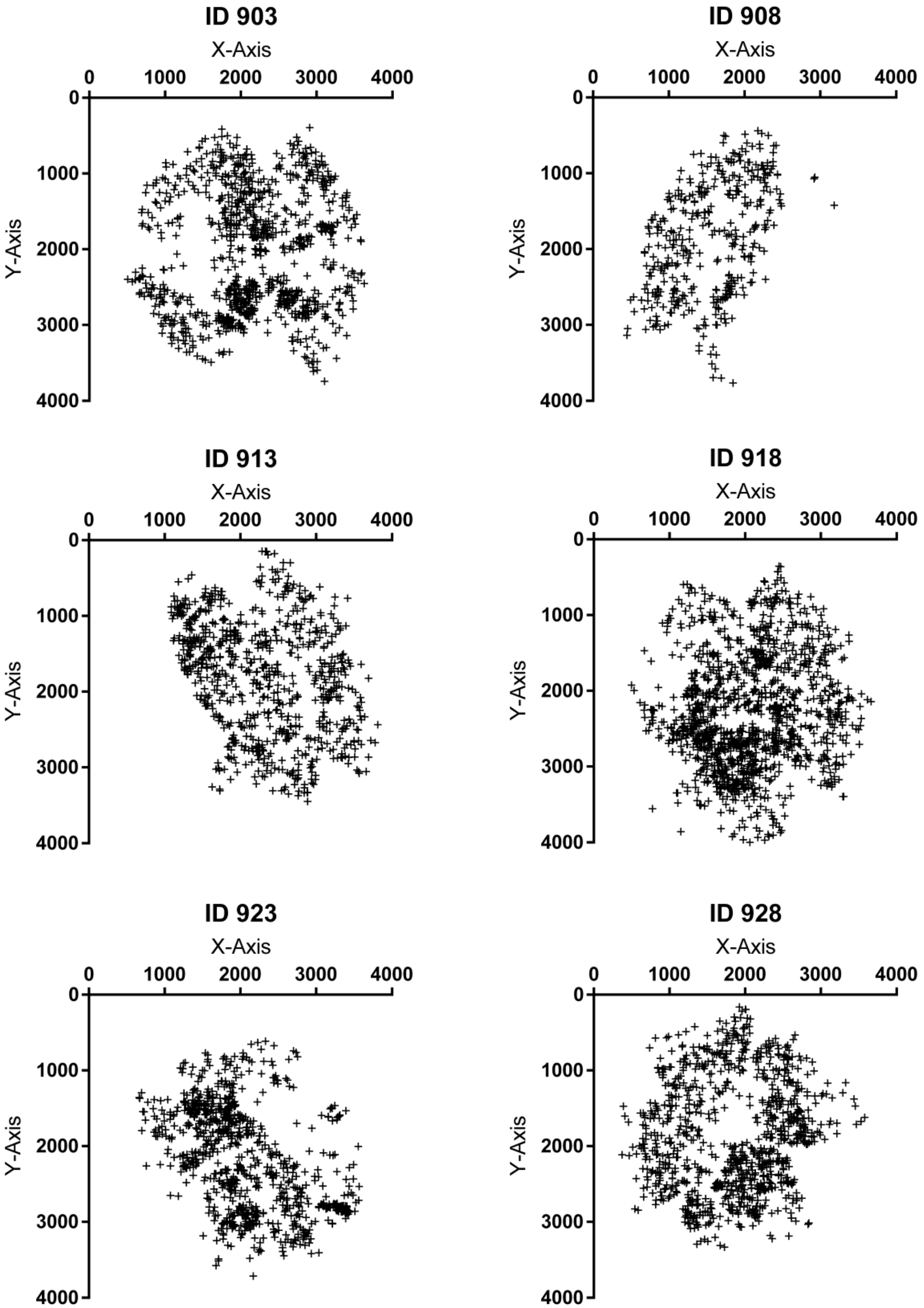

APPENDIX FIGURE S3 - THUMBNAIL IMAGES OF ANNOTATED RETINAL FLAT MOUNTS (TUNEL) AND COORDINATES

C) Group: Anti-VEGF-A

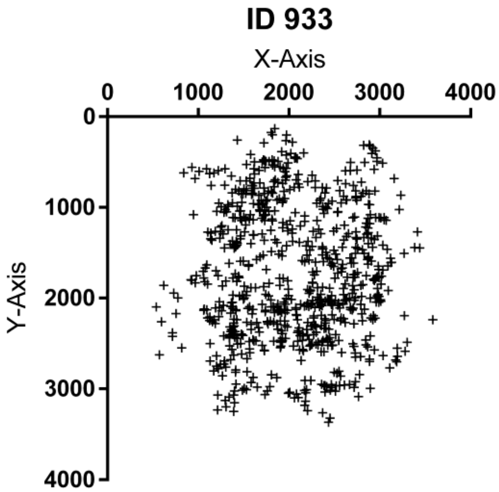

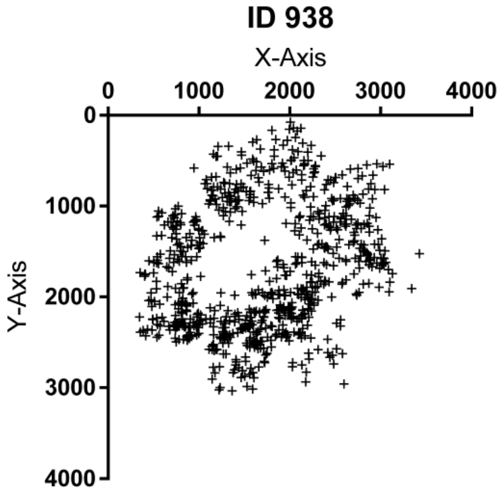

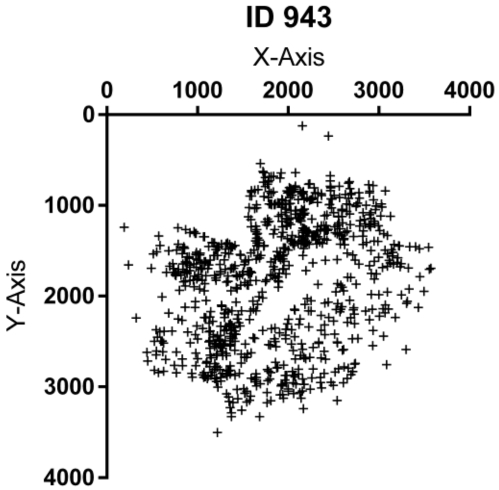

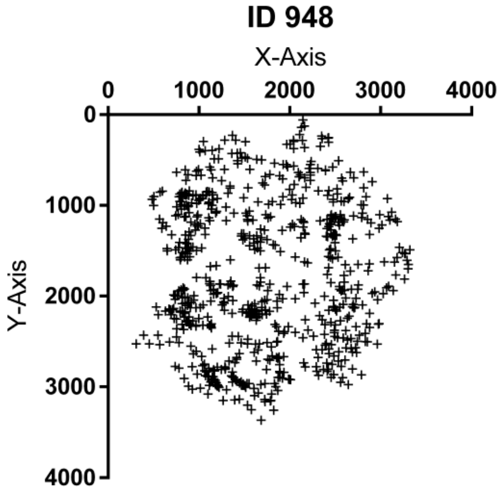

APPENDIX FIGURE S3 - THUMBNAIL IMAGES OF ANNOTATED RETINAL FLAT MOUNTS (TUNEL) AND COORDINATES

D) Group: Anti-ANG-2

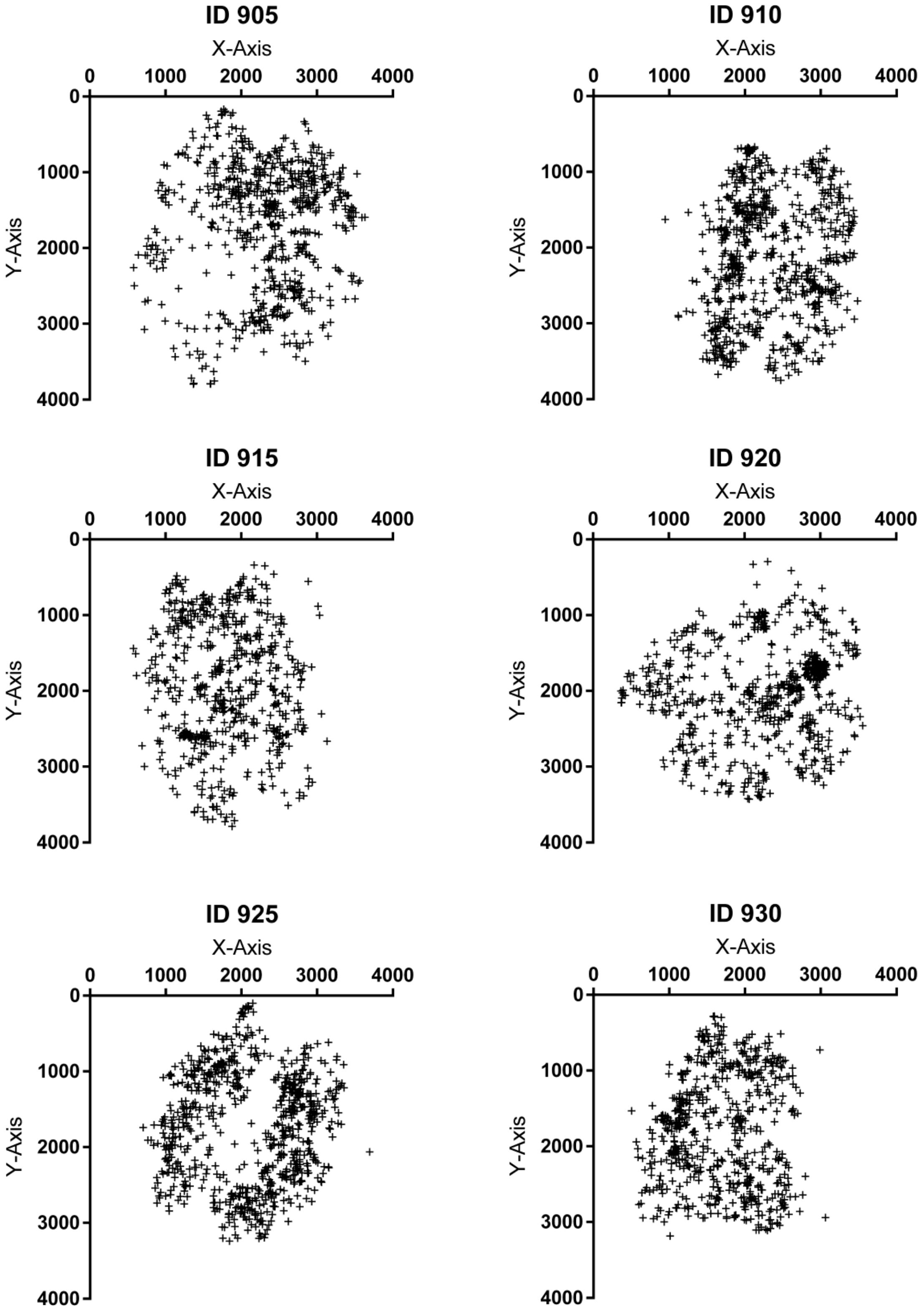

APPENDIX FIGURE S3 - THUMBNAIL IMAGES OF ANNOTATED RETINAL FLAT MOUNTS (TUNEL) AND COORDINATES

D) Group: Anti-ANG-2

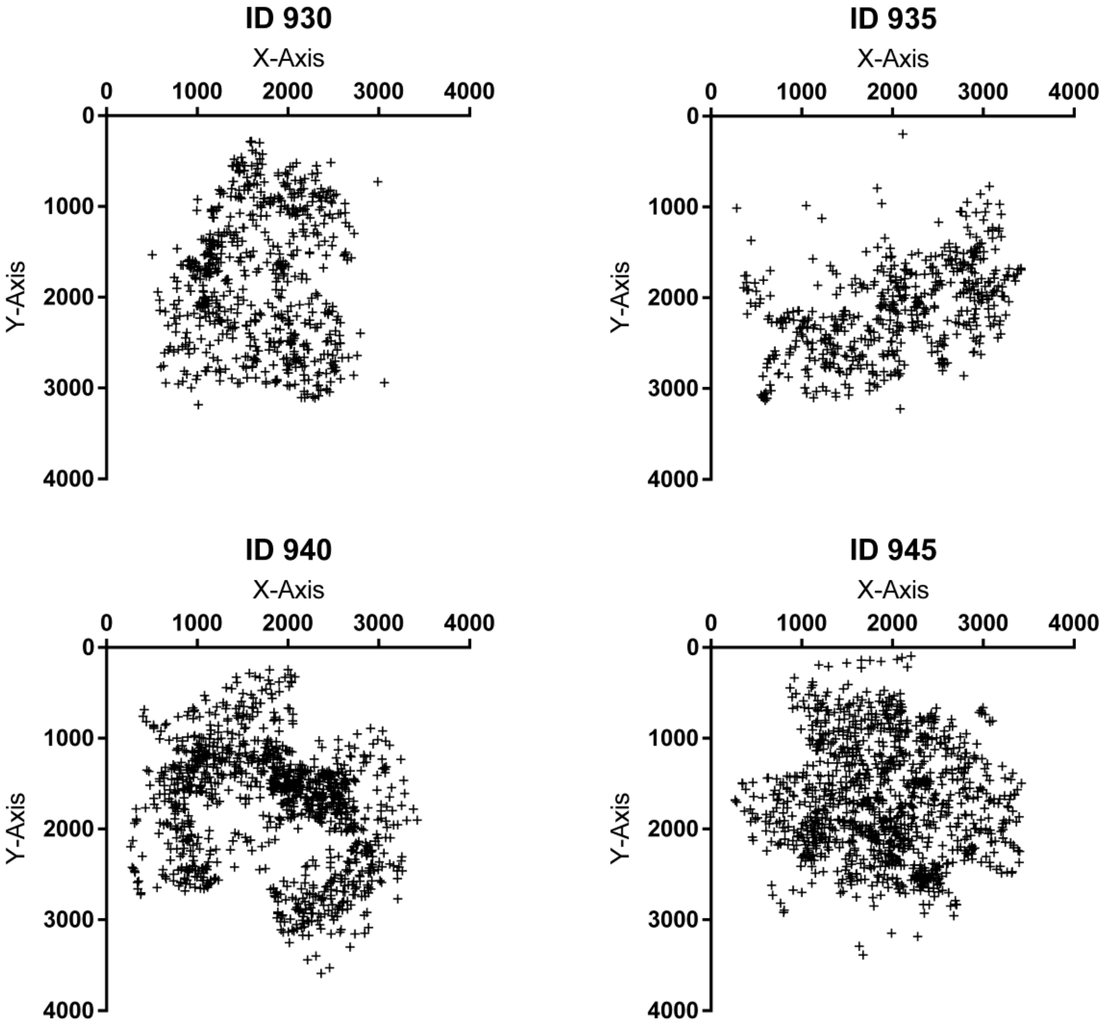

APPENDIX FIGURE S3 - THUMBNAIL IMAGES OF ANNOTATED RETINAL FLAT MOUNTS (TUNEL) AND COORDINATES

E) Group: anti-VEGF-A/ANG-2

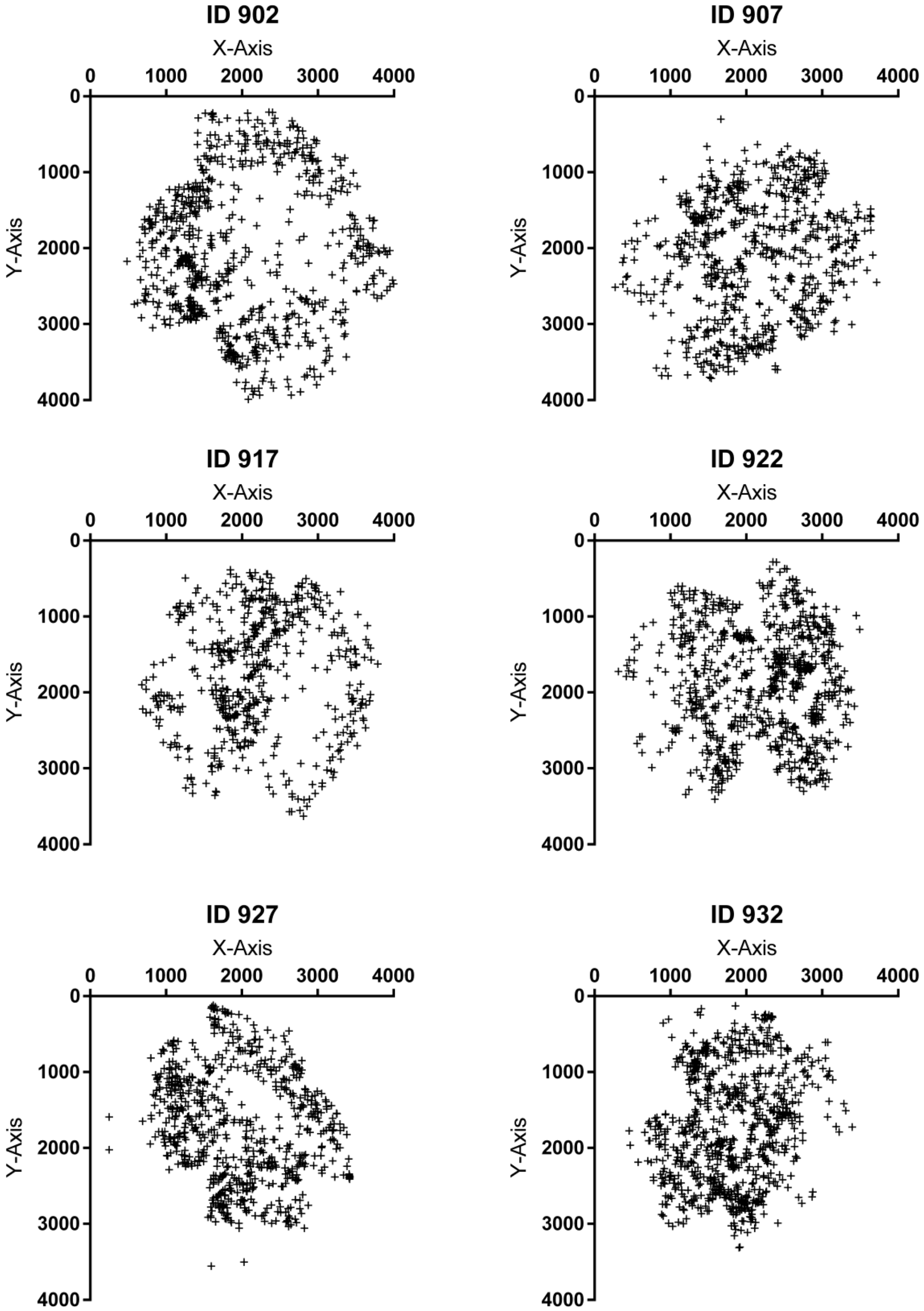

APPENDIX FIGURE S3 - THUMBNAIL IMAGES OF ANNOTATED RETINAL FLAT MOUNTS (TUNEL) AND COORDINATES

E) Group: anti-VEGF-A/ANG-2

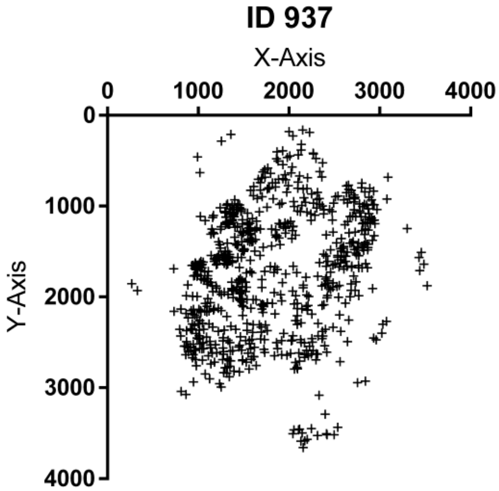

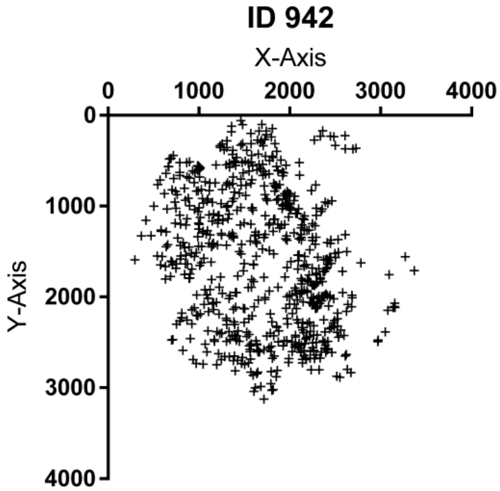

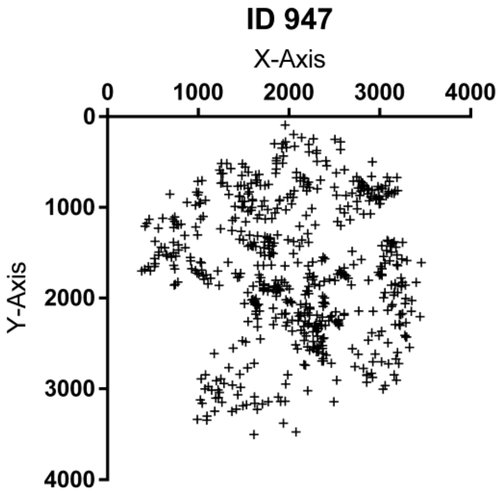

Supplement: Supplementary file 1 — Appendix [file EMMM-11-e10204-s001.pdf]
